# Structure of a barrel-stave pore formed by magainin-2 reveals anion selectivity and zipper-mediated assembly

**DOI:** 10.1038/s41598-025-23539-1

**Published:** 2025-11-13

**Authors:** Enea Sancho-Vaello, Harun Kücükyildiz, David Gil-Carton, Xevi Biarnés, Kornelius Zeth

**Affiliations:** 1 Department of Biochemistry and Molecular Biology, Universitat Autònoma de Barcelona, Building C, Office C2/427, Campus UAB, Cerdanyola del Vallès, 08193 Bellaterra, Spain; 2 Department of Science and Environment, Roskilde University, Universitetsvej 1, Roskilde, 4000 Denmark; 3 Basque Resource for Electron Microscopy, Biofisika, Leioa, Spain; 4 Ikerbasque, Basque Foundation for Science, Bilbao, Spain; 5 CIC bioGUNE, Derio, Spain; 6 Laboratory of Biochemistry, Institut Químic de Sarrià (IQS), University Ramon Llull (URL), Barcelona, 08017 Spain; 7 Faculty of Biology, University of Regensburg, Universitätsstraße 31, 93053 Regensburg, Germany

**Keywords:** Antimicrobial peptides, Structure-function model, Magainin-2, Peptide channels, X-ray structure, Molecular dynamics, Biochemistry, Biophysics, Chemistry, Structural biology

## Abstract

Antimicrobial peptides (AMPs) are ubiquitous weapons of all higher organisms to suppress antimicrobial growth. Despite intensive research, the killing mechanism of these peptides after interaction with the bacterial cell wall and cytoplasm is not well understood. To investigate this mechanism at a molecular level, we chose a well-studied AMP, Magainin-2 (Mag-2), for biophysical and structural studies. Circular dichroism experiments showed that the folding propensity of Mag-2 is strongly altered towards fully folded molecules in the presence of detergent. To study the pore-forming properties of Mag-2 in membranes, we crystallized the wild-type peptide in the presence of the membrane-mimicking dodecylphosphocholine detergent and obtained crystals diffracting to atomic resolution. Mag-2 structure shows an antiparallel arrangement of monomers, which is stabilised by a phenylalanine zipper motif spanning the hydrophobic interaction surface of this dimer. Trimerization of dimers leads to the formation of a hexameric peptide channel complex with a positively charged pore and a hydrophobic membrane-exposed belt. Using molecular dynamics simulations, a spontaneous flow of ions through this channel was observed, demonstrating anion-selectivity induced by the electrostatic potential characteristics of Mag-2. This first atomic-resolution structure of wild-type Mag-2 showing oligomerization will allow the rational design of improved Mag-2 peptide channels.

**Supplementary Information:**

The online version contains supplementary material available at 10.1038/s41598-025-23539-1.

## Introduction

Antibiotic resistance is one of the most pressing global health challenges our world is currently facing. In the European Union alone, multidrug-resistant (MDR) bacterial infections account for over 670,000 cases annually, resulting in approximately 33,000 deaths as a direct consequence^1^. This concerning trend underscores the critical need to develop new bactericidal drugs, whether based on novel chemical scaffolds, traditional antibiotic scaffolds, or natural molecules. Among the natural biological molecules, bacteriophages, endolysins and antimicrobial peptides (AMPs) are the most advanced antimicrobials^2–5^. In the case of artilysins, AMP-fused endolysins, AMPs play a crucial role in breaking the otherwise highly protected Gram-negative bacterial cell wall^6^. Despite the vast number of AMPs described in the literature, only around 40 AMPs are currently in preclinical or clinical trials, most of which target topical infections with approximately 10 candidates in phase III clinical studies^7–9^.

AMPs are part of the innate immune system playing crucial roles in combating multi-drug resistant pathogens, inflammation, angiogenesis and wound healing^10,11^. The ~ 5099 AMPs identified to date, encompassing both natural and synthetic variants, share a surplus of positive charges as a common feature, which enables their interaction with negatively charged bacterial surfaces^12^. However, their structural and functional diversity prevents a single, universal killing mechanism from being defined. AMPs typically interact with the negatively charged and cell surface-exposed lipopolysaccharide (LPS) or lipoteichoic acid (LTA) molecules in Gram-negative and Gram-positive bacteria, respectively, as first point of contact^13,14^. Beyond this, AMPs can target outer membrane proteins, the periplasmic space and in particular cytoplasmic membrane lipids, often referred to as the bacterial cell´s ‘Achilles heel‘^15^. AMPs that successfully cross the cytoplasmic membrane may also interact with cytoplasmic proteins, DNA, or RNA, disrupting essential cellular functions^16,17^. Upon interaction with membranes and other intracellular targets, AMPs usually undergo secondary structure changes, often assembling into oligomers, fibrils or fibres^18–23^. Oligomeric states at membranes and membrane-mimics allow AMPs to increase their local concentration, facilitating the formation of membrane-disrupting structures that ultimately lead to bacterial cell death. Three basic models describing the interactions of -helical amphipathic AMPs with model membranes have been developed^15,24,25^. The carpet model assumes the disruption of the membrane in a detergent-like manner, with AMPs encapsulating lipid micelles^26^. The toroidal model describes the pore formation by AMPs strongly interacting with both lipid head groups and alkyl chains assuming minimal peptide-peptide interactions^27–30^. The barrel-stave model outlines the assembly of amphipathic peptides into structures of defined transmembrane pores, due to strong intermolecular peptide-peptide interactions, with their hydrophobic regions exposed to the membrane environment^31^.

The barrel stave model which describes a rigid peptide complex disrupting membranes is possibly the most well-defined mechanistic model, supported by X-ray data. However, up to date, there are only two natural AMPs, the human dermcidin (DCD) and LL-37, which have been structurally validated at atomic resolution as forming defined transmembrane channels^18,23^. The DCD structure, solved by X-ray crystallography, shows a highly charged hexameric channel (trimer of dimers) of 8 nm length and approximately 1 nm inner diameter stabilised by zinc ions, which introduce discrete conductance steps in planar membranes^18,32,33^. Although the structure was solved in the absence of detergents and lipids, the channel clearly shows the archetypical features of six membrane-spanning helices arranged in pairs of anti-parallel dimers with a hydrophobic surface facing the putative membrane^18^. More recently, we also succeeded in the determination of a tetrameric LL-37 channel (a dimer of dimers), which shares similar structural properties with DCD^23^. Another well-known channel-forming AMP is alamethicin, which has served as a paradigm for a voltage-gated peptide channel. *Woolley et al.* initially suggested that alamethicin functions as a dimer^34^, but later, a hexameric channel structure was proposed based on atomic force microscopy (AFM) studies^35,36^. This hexameric assembly was further supported by electrochemical scanning tunnelling microscopy (EC-STM), which imaged hexameric pores formed in a phospholipid matrix^37^.

Several molecular dynamics (MD) simulations have also been performed on primarily monomeric AMPs, exploring their potential assembly on membranes, their location within membranes, and their ability to form pore structures^22,38,39^. These simulations have revealed that not only does the membrane´s composition influence the conformational state of the peptides, but it also affects the likelihood of the peptides inserting into the membrane´s interior. Some other recent simulations investigated the role of Pro/Gly kinks in AMPs, suggesting that these kinks promote the formation of toroidal pores rather than barrel-stave pores^40^. Furthermore, MD simulations of the hexameric DCD channel demonstrated its stability within a phosphatidyl ethanolamine/phosphatidyl glycerol (POPE/POPG, 3:1) membrane system, and the conductivity properties of the channel were confirmed by planar lipid membrane experiments^18^. In these works, the choice of the membrane system was found to be crucial in determining the conductivity^41^. Similar stability and conductivity calculations were also performed for the narrow tetrameric LL-37 channel, but the simulations did not observe ion translocation, even though conductivity experiments confirmed the channel´s formation in an artificial membrane^23^.

Magainin-2 (Mag-2) is a well-studied AMP, originally isolated from frog skin, consisting of 23 residues and carrying a net positive charge of + 3 (sequence: GIGKFLHSAKKFGKAFVGEIMNS)^42^. It has been shown to effectively kill MDR strains of *Escherichia coli*, *Klebsiella pneumoniae*,* Pseudomonas aeruginosa* or *Staphylococcus aureus*^42^. Mag-2, along with some derivatives such as pexiganan, is currently undergoing clinical trials for use as a topical anti-infective agent, particularly in the treatment of diabetic foot ulcers^7,8^. Like other AMPs, Mag-2 interacts with negatively charged components such as LPS, LTA, and bacterial cytoplasmic membranes, leading to membrane destabilization. Antimicrobial variants of Mag-2 have been developed, with a truncated 17-residue peptide (H-GIGKFLHSAKKFGKAFV-NH2) demonstrating equivalent antimicrobial activity to the wild-type peptide^43^. Due to the rather loose folding properties of Mag-2 carrying four glycine residues, both natural and non-natural residues have been introduced to enhance its α-helicity^44^. A well-known synergistic combination of AMPs is PGLa (GMASKAGAIAGKIAKVALKAL-NH2) and Mag-2, both found in the skin of the African frog *Xenopus laevis*. Truncated peptides where the Gly residues are exchanged against Lys and Leu showed a tenfold activity increase^43^. Furthermore, *Patch and Barron* reported antimicrobial peptides as Magainin-2 mimetics, showing that a minimum peptide length of approximately 12 residues is required to retain antimicrobial activity^45^.

Even though it has been more than 25 years since the monomeric structure of wild-type magainin was resolved by NMR in the presence of DPC and SDS micelles, no high-resolution crystal structure of the wild-type Mag-2 peptide has been achieved to date. In addition, the killing mechanism of Mag-2 remains unclear^46^. In the NMR structure, the -helix exhibits a curved conformation, with a bent at residues Phe12 and Gly13. Studies have also explored modified Mag-2 peptides, such as fusions with portions of cecropin or melittin sequences, some of which showed similar helicity in DPC micelles^47,48^. Additionally, disulfide-linked Mag-2 peptides, including two additional point mutant analogues, have been analysed by NMR and were found to form an antiparallel peptide dimer. This peptide also formed a supramolecular pore structure in the presence of lipids, as confirmed by circular dichroism (CD) and fluorescence analysis^49^. Recent crystal structures of modified Mag-2 peptides were reported for racemic Ala-(8,13,18) Mag-2 and Ala-Mag-2 with a beta amino acid substitution at position 8 or 13. These peptides showed structurally similar antiparallel dimers^50,51^.

Structures of AMPs can be important snapshots for our understanding of their function and mechanism. We hypothesized that wild-type Mag-2 forms stable oligomeric pores under membrane-mimicking conditions, enabling chloride-selective transport. Therefore, we set out to investigate wild-type Mag-2 in more detail and focused on the functional mechanism in the presence of detergents mimicking membrane permeabilization states. The presence of detergents at concentrations higher than the critical micelle concentration (CMC) significantly altered the secondary structure properties of Mag-2. CD was used to identify the optimal detergent concentration for crystallization. Through this approach, we obtained a hexameric channel structure of Mag-2 (trimer of dimers) at atomic resolution, with hydrophobic residues facing the exterior and positively charged residues oriented towards the pore´s interior. The structure was subjected to molecular dynamics simulations to assess its stability within membrane models and to evaluate its ion permeability, comparing this finding with existing data from planar lipid membrane measurements. The simulations confirmed the stability of the channel within a POPE/POPG (3:1) membrane and provided estimates of anion selectivity and ion flow, supporting our initial hypothesis. Additionally, cryo-electron microscopy (cryoEM) images allowed the visualisation of the membrane disruption induced by Mag-2. This work represents the first atomic-resolution crystal structure of wild-type Mag-2 showing a hexameric assembly, extending beyond previous NMR studies that revealed monomeric molecules and crystallographic studies of Mag-2 with racemic residues that showed dimers but were lacking higher-order organization. The new structural and biophysical data provides the basis to design Mag-2 variants with enhanced properties, such as increased stability, foldability, channel forming capacity and improved antimicrobial activity, which are often linked to secondary structure and oligomerization. While the Mag-2´s activity has been demonstrated in clinical studies, new Mag-2 designs could serve as a new starting point for the generation of improved molecules for future patient studies.

## Results

### The secondary structure content of Mag-2 is strongly dependent on DPC concentrations

To identify the optimal conditions that promote the formation of the α-helical entity and the subsequent assembly of higher order oligomers, the secondary structure of Mag-2 was determined using circular dichroism (CD) in the presence and absence of the detergent dodecylphosphocholine (DPC). In a physiological buffer (10 mM phosphate, pH 7), Mag-2 exhibited a strong negative band around 203 nm and a negative shoulder at 225 nm, which are characteristic of a random coil structure (see Fig. S1)^52–56^. This pattern changed after the addition of the detergent DPC. Mag-2/DPC micelles were analysed at increasing DPC concentrations starting below the critical micelle concentration (CMC_DPC_: ~1.5 mM; ~ 0.05%) (see Fig. S1). At the lowest DPC concentration, the spectrum was similar to the spectrum observed in the absence of DPC. However, as DPC concentration increased to 1.4 mM (0.05%, P: L ratio 1:35) or 5.7 mM (0.2%, P: L ratio 1:142), the spectra displayed minima at 208 and 222 nm, along with a positive maximum at 195 nm, indicative of the formation of an α-helical conformation (see Fig. S1)^57^.

### Mag-2 forms an antiparallel dimer stabilized by a phenylalanine zipper motif

In the past, we analysed the functional properties of various AMPs, focusing on their ability to form channels in planar lipid membranes. We hypothesized that the presence of defined conductance steps for AMPs in planar lipid membranes, along with the formation or prediction of an amphipathic α-helix, could indicate the potential for channel formation by the peptide, likely via the barrel stave mechanism. The formation of a defined pore structure suggested that the peptide can maintain this structure, making it suitable for further analysis through crystallography. To test this hypothesis, we previously chose Dermcidin, and LL-37^18,23^. In this paper, we focus on Mag-2 which had previously also been shown to exhibit discrete conductivity steps when embedded in planar lipid membranes^58^.

**Fig. 1:**
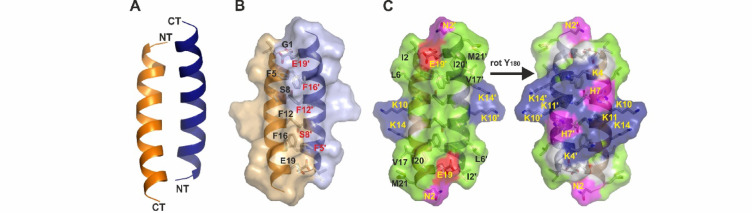
The dimeric Magainin-2 structure shows a hydrophobic interface and two surfaces with opposite properties.** (A)** The monomeric Mag-2 structures including residues Gly1 to Met21 form a slightly bent anti-parallel helix dimer shown in ribbon representation with the termini are marked with NT and CT (N-terminus, C-terminus respectively). **(B)** Surface representation of the two monomers with the hydrophobic interface residues labelled according to their sequence positions. **(C)** Surface representation of Mag-2 in the same orientation as (A) with hydrophobic residue positions colored in green, negatively charged residues in red, positively charged residues in blue and hydrophilic uncharged residues in magenta. The dimer is shown from the two opposing sides demonstrating the distribution of hydrophobic residues towards one and hydrophilic residues towards the other side.

Mag-2 is one of the most extensively studied AMPs, with a substantial amount of functional data available in the literature and numerous applications in biopharmaceuticals^28,59–61^. To better interpret these data, including various planar lipid membrane conductivity studies, we conducted crystallisation trials of Mag-2 both in the absence and presence of 0.2% DPC and 0.2% n-dodecyl-beta-maltoside (DDM) detergents. The use of detergents was intended to stabilise the peptide´s secondary structure, as demonstrated by circular dichroism for DPC (see Figure S1). While we failed in generating diffracting crystals in the absence of detergents, we successfully obtained crystals in the presence of 0.2% DPC (5.6 mM), but not in the presence of DDM. These crystals diffracted to 1.05 Å resolution, and the structure was solved by molecular replacement using the monomeric NMR structure (PDB-entry: 2MAG) as the search model. One monomer was observed in the asymmetric unit. The structure of the monomer displayed a single α-helix comprising all residues (Gly1-Asn22) except Ser23, which was not ordered. The helical part included residues Ile2 to Met21 (see Fig. 1A). Dimerization occurred with a second symmetry-related monomer of the crystal packing and the resulting interface of 510 Å^2^ showed a clear biological significance (biological score of 1 when analysed by the program package PISA^62^. The interactions between the two monomers were mediated by hydrophobic residues including Phe5, Phe12, Phe16 and alanines 9 and 15 (see Fig. 1B). Notably, the aromatic side chains formed a long-range connected network of five aromatic interactions arranged in a phenylalanine zipper (Phe5/Phe16’, Phe5/Phe12’, Phe12/Phe12’, Phe12/Phe5´, Phe16/Phe5’; see Fig. 1B and S2, and Table S1). The dimer exhibited two electrostatically different interfaces, as shown in Fig. 1C. One interface was strongly hydrophobic, with six phenylalanines Phe5/Phe5’, Phe12/Phe12’ and Phe16/Phe16’ oriented along the same direction of the dimeric symmetry axis, alongside additional aliphatic side chains at the N-and C-terminal ends (See Fig. S2). The opposite side of the dimer was dominated by hydrophilic residues, including eight lysines (Lys4/Lys4’, Lys10/Lys10’, Lys11/Lys11’ and Lys14/Lys14’), two histidines (His7/His7’) and two glutamates (Glu19/Glu19’) at the C-terminal end of the peptide contributing to an overall charge of + 6 (see Fig. 1C).

### Three Mag-2 dimers assemble into a hexameric pore structure with a positively charged channel interior

**Fig. 2:**
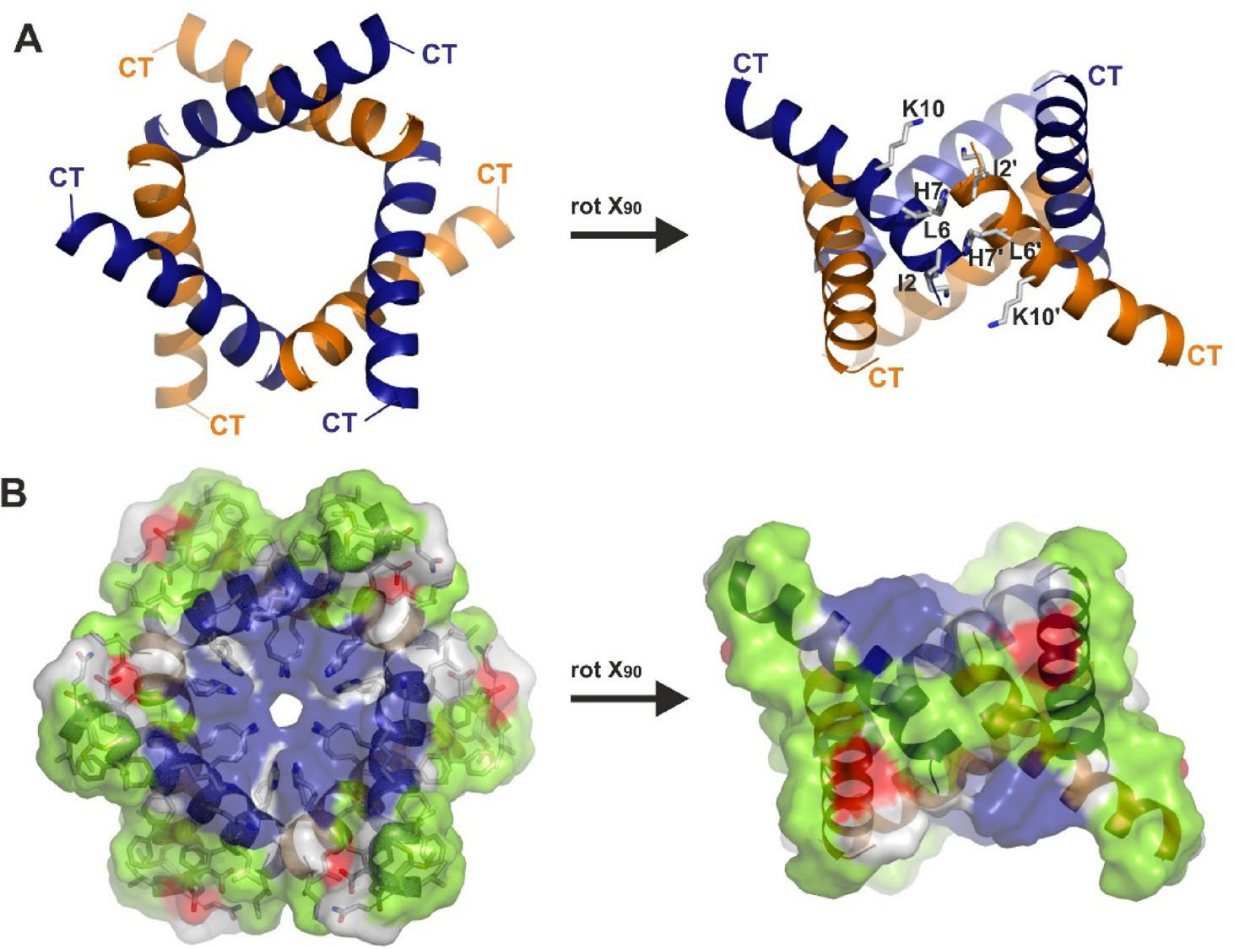
Trimers of Mag-2 dimers form a hexameric structure with the hydrophobic interface exposed towards the membrane and a positively charged channel facing inward. **(A)** The hexameric Mag-2 channel structure depicted in cartoon representation, assembled via trimerization of dimers (NT: N-terminus, CT: C-terminus). Two orientations of the structure are shown as top and side-view related to each other by a rotation of 90 degrees around the X-axis. One of the dimers forms a wall-like structure facing the membrane surrounding it. **(B)** Surface representation of the two hexameric structures shown in the same orientation as (A) and color-coded based on the amino acid residual properties. Hydrophobic residues are colored in green, positively charged residues are blue and negatively charged residues are red and amphiphilic residues in magenta.

Upon further examination of the crystallographic environment of the dimer, we noticed that a hexameric channel-like structure based on three peptide dimers and threefold symmetry with reasonable peptide-peptide interfaces could be constructed (PISA´s resulting interface of 4596 Å^2^). The outer overall dimensions of this hexameric channel were 3.5 nm in height and 4.5 nm in width, and a very narrow central pore of approximately 0.35 nm (see Fig. 2). The dimer-dimer interface was stabilized by a combination of hydrogen bonds and hydrophobic interactions, contributed by the Mag-2 N-termini. This interface was smaller than the monomer-monomer interface discussed in the previous paragraph. The pore formed by these peptides exhibited a slightly uneven height, with all positively charged residues facing inward. The hydrophobic belt facing outward contained essentially the same residues as the hydrophobic dimer face. Within the pore, two layers of adjacent positive charges, mainly Lys10 and His7, were located, while residues 10–22 did not significantly contribute to the inner pore architecture.

### Molecular dynamics simulations demonstrate Mag-2 channel stability in POPE/POPG membranes

**Fig. 3:**
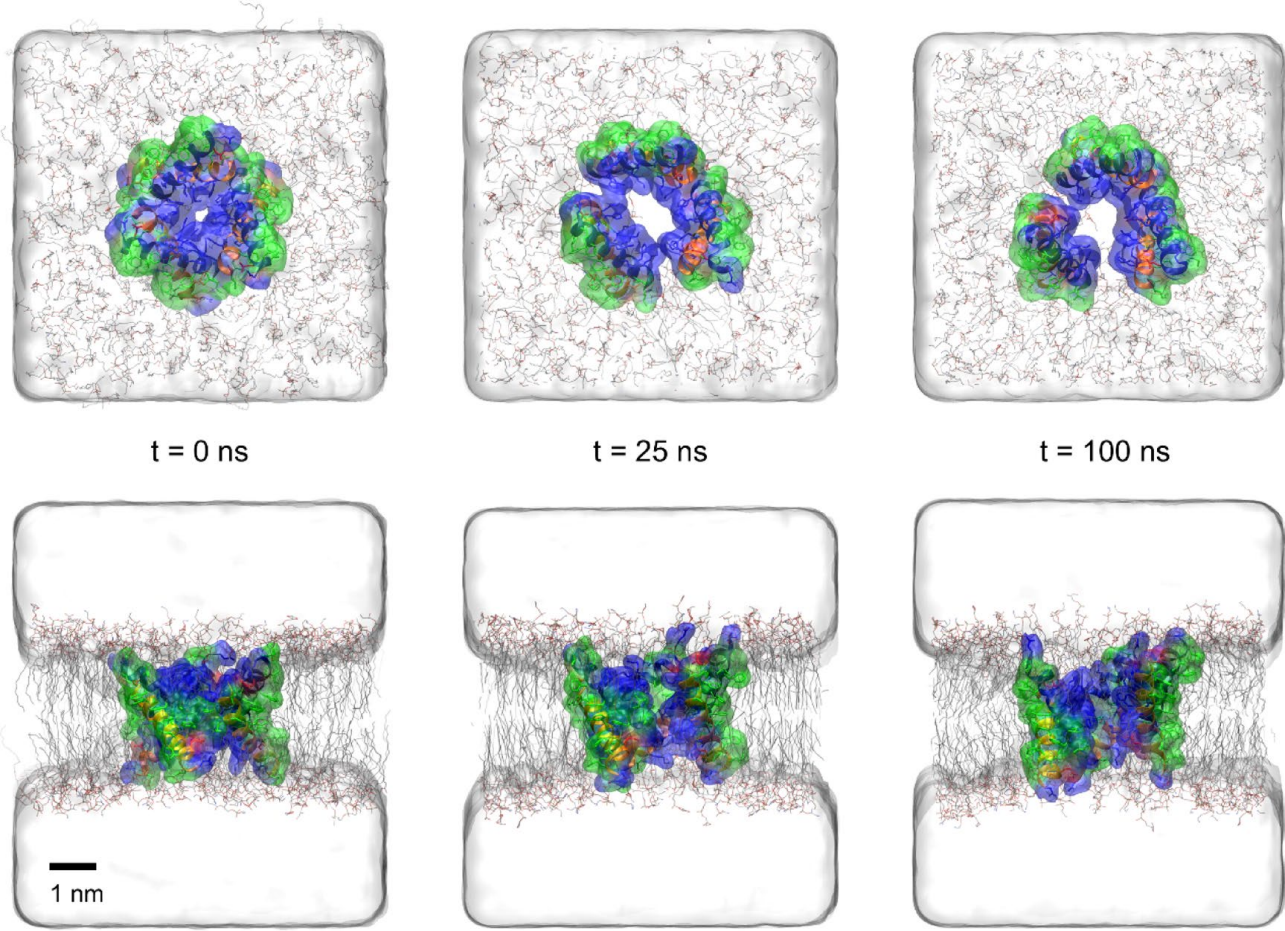
Evolution of the magainin-2 channel along the simulation (at time 0 ns, 25 ns, and 100 ns). First row: side-view; second row: top-view. The hexameric structure is shown in ribbons and solvent accessible surface colour coded according to the amino acid properties as in Fig. 2B. Membrane lipids are represented as lines and coloured by atom type (C: black, O: red, N: blue). Solvent is represented as a white cubic volume. Explicit water molecules and ions, present during the simulation, are omitted for clarity.

The stability of the hexameric Mag-2 channel in a physiological membrane and the formation capacity of a permeable pore was assessed by molecular dynamics simulations. The hexameric Mag-2 structure was embedded in a POPE/POPG membrane bilayer (3:1 ratio composition) as done in^23^ (see Fig. S3). The initial orientation of the channel was perpendicular to the membrane plane (see Fig. 3, t = 0 ns). The whole system was simulated by means of all-atom molecular dynamics in explicit solvent and K^+^ and Cl^−^ ions at 1 M concentration placed to the volumes adjacent to both sides of the membrane (see methods). The structure of the channel was overall stable within the POPE/POPG membrane, as seen by the snapshots along the simulation (see Fig. 3) and the RMSD evolution of the entire hexamer (see Fig. S4A). Nevertheless, some subtle rearrangement of the initial structure occurred. The individual dimers were slightly tilted vertically to better accommodate the polar amino acids at the N- and C- terminus (Lys4, Asn22) with the polar head groups (phosphates and amines) of the lipids (see Fig. S5). In particular, two of the dimers got separated resulting in an opening of the channel. The gyration radius of the hexamer increased 9% with respect to the initial structure and got stabilized at an average value of 1.74 nm (see Fig. S4B). As a result, the tiny pore initially observed in the crystal structure of the isolated hexamer expanded to a channel of diameter 0.70 nm. This allowed the flow of water molecules and ions through the pore, which was hampered in the compacted initial hexameric structure.

### Chloride anions flow along the Mag-2 hexameric channel

**Fig. 4:**
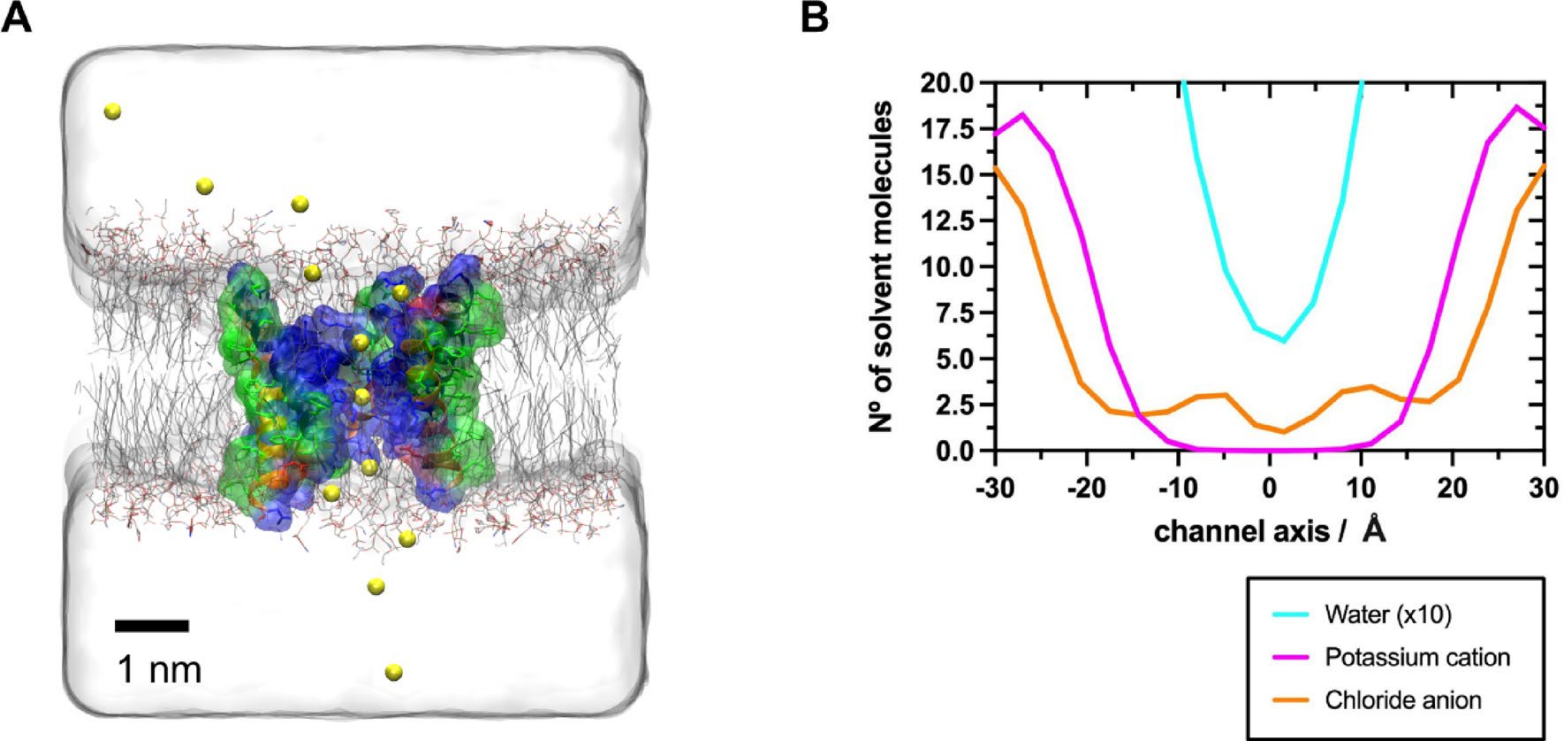
**(A)** Example of a chloride anion (yellow sphere) passing through the hexameric magainin-2 channel at different times of the simulation. Peptides are represented and colored as in Fig. 3. Membrane lipids are represented as thick lines and coloured by atom type (C: black, O: red, N: blue). Solvent is represented as a grey cubic volume. **(B)** Average density of solvent molecules (water, potassium cation and chloride anion) across the principal channel axis during the whole simulation. For an explicit representation of the density, see Figure S6.

Molecular dynamics simulations were carried out in explicit solvent with atomistic detail, revealing the flow of water molecules and Cl^−^ and K^+^ ions across the different parts of the system. Five independent replicates were simulated for 100 ns each replica. All replicates showed similar behaviour. A movie illustrating the simulation of one representative replica can be found in the supplemental material. Water and ions freely move around the POPE/POPG membrane bilayer, as well as both the head and tail of the Mag-2 hexameric channel. The inner part of the channel is continuously solvated by waters. As long as the initial pore is expanded, the simulation reveals chloride anions freely passing through the channel. It should be noted that no external potential was applied to force this anionic flow. As an illustrative example, Fig. 4A shows the path followed by one chloride ion during a short period of the simulation time (for a border picture, the path followed by all Cl^−^ and K^+^ ions along the whole simulation is illustrated in Fig. S6). Since periodic boundary conditions are applied to the system (see methods), there is not any charge accumulation at either side of the membrane. Ions and water molecules passing through the pore are free to move back to the other side of the membrane via the periodic boundary, maintaining an equal overall concentration of each solvent species.

The average density of water molecules, Cl^−^, and K^+^ ions along the channel axis was computed for the whole simulation (see Fig. 4B). Outside the channel, there are around 15 Cl^−^ and 18 K^+^ ions on average above the membrane plane. Inside the channel, an average of 17 Cl^−^ ions pass through the entire length of the channel, with one chloride anion in the centre of the channel solvated by 7–8 water molecules. Interestingly, the channel is not permeable to potassium cations. These cations accumulate at the entrance of the channel and along both sides of the membrane surface (higher K^+^ ion densities compared to Cl^−^ ions), but cations are unable to pass through it. The density of K^+^ ions observed in the channel is essentially zero along the entire channel) (see Fig. 4B). These findings align with the specific nature of the Mag-2 hexameric channel, which is rich in lysines and histidines in the inner part of the pore, while the only negative amino acid (glutamate) is located at the external part. These Mag-2 pore characteristics generate a significant positive electrostatic potential inside the channel (see Fig. S7). The Mag-2 hexamer creates a dense electrostatic field from the solvent that passes through the channel, providing a pathway for anion migration. In this system configuration, the potential difference across the membrane is + 0.6 V (see Fig S7). Consequently, Cl^−^ ions spontaneously flow through the Mag-2 hexameric pore, at a rate of 54 Cl^−^ ions every 100 ns. The average time for a Cl^−^ ion to pass through the entire channel is 4.1 ± 2.7 ns. Thus, the average channel conductance can be estimated to be 0.9 nS, which is in the same range as experimental measurements done with magainin-1 (a closely related peptide)^63^.

### Electron microscopy analysis of ***E. coli*** cells treated by Mag-2

**Fig. 5:**
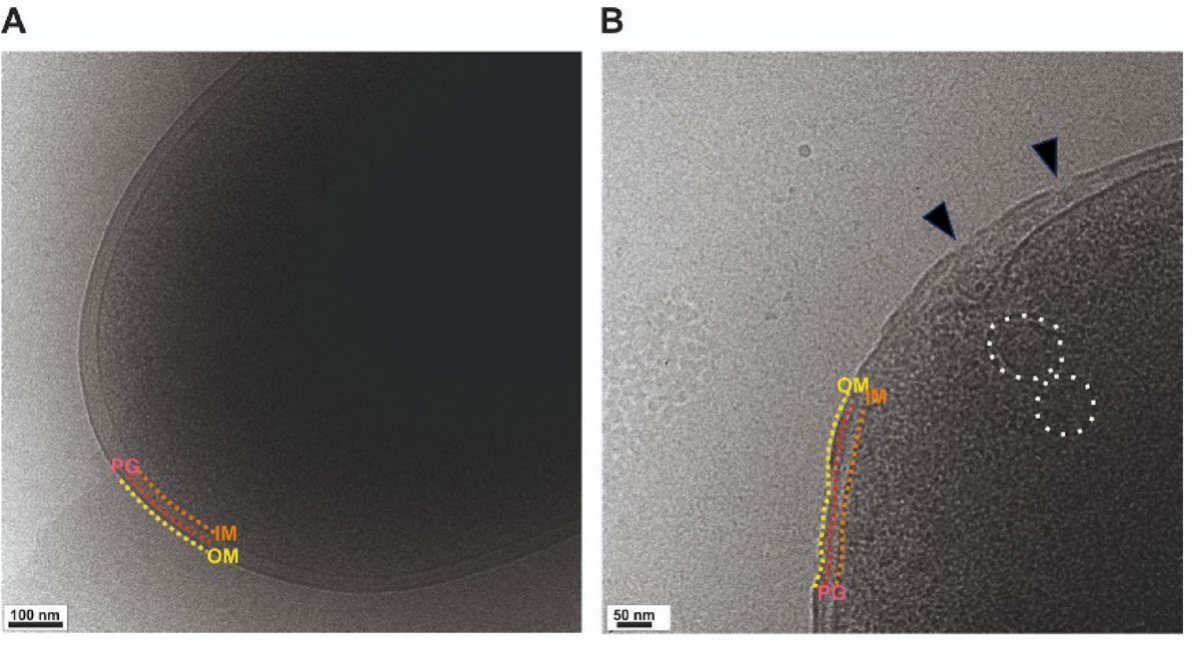
Cell wall disruption of E. coli cells induced by Mag-2 imaged by cryo electron microscopy. (**A**) *E. coli* K12 cells were imaged in the absence of Mag-2 and show an undisturbed cell wall with outer membrane (OM - marked in yellow), peptidoglycan (PG - marked in red) and inner membrane (IM - marked in orange) clearly separated and undisturbed. The scale bar shows the dimension of 100 nm. (**B**) *E. coli* K12 cells were incubated with Mag-2 at concentrations of 25 µM slightly above the MIC and imaged under the same conditions as the untreated cells. Here, the three layers are no longer undisturbed but show serious changes (the three layers are marked in the same way as in (A)), in particular the outer membrane shows clear hole-like structures (see black triangles). The inner membrane shows similar disruptive features and vesicles derived from inner membrane lipids are indicated with white circles.

The initial interaction of AMPs with Gram-negative bacteria is mediated by surface exposed and negatively charged structures of the cell wall, mainly lipopolysaccharides (LPS). In order to elucidate the mode of interaction of Mag-2 with the cell wall and identify which of the three main levels of the cell wall, the outer membrane, the peptidoglycan and the inner membrane might be most affected, we followed the same approach as used in^23^. Briefly, K12 *Escherichia coli* cells were exposed to 25 µM Mag-2 (MIC ~ 50 µM^64^) for 1 h, and the disruption mechanisms were observed using cryo-electron microscopy. When imaging *E. coli* K12 without Mag-2, the three layers were clearly visible as separate entities (see Fig. 5A). Upon exposure to Mag-2, significant damage to the cell wall was observed. While the outer and inner membrane remain distinguishable in some areas, the outer membrane exhibited clear 10–50 nm gaps, indicative of hole-like structures (see black triangles in Fig. 5B and S8). While the formation of some of these was not evident in every cell, discontinuities or holes were visible in many cells (see Fig. S8). The outer membrane also became notably corrugated, as opposed to its smooth appearance in untreated. The peptidoglycan layer showed less damage, but in some regions, it was not clearly visible. The inner membrane showed the formation of stress vesicles and other signs of significant disruption. Lastly, the cytoplasm displayed a very homogeneous distribution of densities in the absence of Mag-2, but after incubation with the peptide, condensation occurred, along with the formation of larger, denser particles.

## Discussion

The emergence of multidrug-resistant bacteria requires new antibiotic strategies, including the development of novel molecular classes. A battery of biological molecules including phages, endolysins, artilysins, antibodies, and antimicrobial peptides (AMPs), are currently undergoing clinical studies, though with varying degree of success^65–67^. Around 40 AMPs are currently in various phases of clinical trials, yet none of them has been approved for routine clinical use^7–9^. This limited success is largely attributed to the lack of clear efficacy advantages over conventional antimicrobials, which remain effective and inexpensive despite contributing to the rise of antimicrobial resistance. Because AMP-based therapeutics are generally less prone to resistance development, clinical trials often employ equivalence or non-inferiority designs when assessing AMPs efficacy^68^. One of the AMPs of particular interest in this context is pexiganan (MSI-78), a synthetic analog of Mag-2 which has shown potential as a topical treatment for diabetic foot ulcers^69^. Although pexiganan reached phase III clinical trials (CT identifier: NCT01590758, NCT01594762), it did not receive FDA approval likely due to issues related to peptide stability, suboptimal drug administration, or interactions with standard treatments (notably, for ethical reasons, clinical trials often administer both the placebo and investigational drug in combination with the standard care)^70^. This is why the formulation and clinical application of pexiganan are currently under revision^9^.

Traditionally, X-ray structures of small molecule antibiotics bound to their targets, such as ribosomes or β-lactamases, have significantly advanced the rational design of new antibiotics^71–75^. By using a similar approach, structures of AMPs in complex with their targets such as lipopolysaccharide (LPS) or membranes will be highly beneficial to engineer novel peptides with enhanced activity. However, AMP-target interactions are inherently more complex, as these peptides can interact with multiple cellular targets (e.g., membranes, LPS, DNA)^13,14^ rather than a single, well-defined target. This complexity is largely driven by their highly cationic nature, which facilitates broad-spectrum interactions.

In this work, we aimed to investigate the structure and function of Mag-2, which had never been successfully crystallized as wild-type peptide. A major obstacle to crystallization appears to be the intrinsically disordered nature of Mag-2 in aqueous solution. However, in the presence of detergents at or above their critical micelle concentration (CMC), the peptide adopts a fully α-helical conformation, suggesting that membrane-mimicking environments are required for its structural stabilization. To improve the likelihood of successful crystallisation, we employed CD to determine the DPC concentration required to induce maximum folding of Mag-2. Our results indicated that 0.2% DPC was the optimal concentration for folding the peptide, aligning with similar findings by others showing that Mag-2 is unfolded in buffer but well folded in the presence of detergents, LPS or *E.coli* cells^54^. The ability of AMPs to fold in response to detergents has also been shown for LL-37^23^ and anoplin^76^.

In the case of LL-37, our group has previously demonstrated direct atomic-level interactions between the detergents DPC and LDAO, offering a structural basis for how detergents promote the folding and stabilize the conformation of AMPs^20^. While we failed to obtain crystals of Mag-2 in the presence of DDM, we successfully achieved crystallisation in the presence of a Mag-2:DPC ratio of 1:3.5 (4 mM Mag-2 and 0.5% DPC = (14 mM)). These crystals diffracted to atomic resolution, revealing a α-helical three-dimensional structure in which two antiparallel monomers associate to form a dimer via a prominent hydrophobic interface (see Fig. 1). The dimerization of Mag-2 is primarily driven by a phenylalanine zipper motif, which plays a critical role in stabilizing the dimer (see Fig. 1 and S2). This motif was shown to be essential for folding in a Mag-2 variant, in which deletion of two phenylalanine residues led to disassembly of the dimer even in the presence of detergents^77^. In the context of this paper, future work will include structure-guided mutagenesis with double and triple mutations targeting the zipper motif (F5A, F12A, F16A) and pore-lining residues (K10A, K11A, H7A) to validate the necessity of pore formation for bactericidal activity.

Antiparallel dimerization appears to be a common feature among AMPs, including LL-37, melittin and dermcidin (DCD), which form dimers capable of further oligomerization (Fig S12 in^23^). These AMP dimers typically exhibit amphipathic surfaces, with one side exposing hydrophobic residues and the opposite side exposing a hydrophilic surface^18,20,23^. While this amphipathic organization is conserved across AMP dimers, their size relative to membrane dimensions varies depending on the number of residues of the peptide. In this sense, the largest structurally characterized AMP, DCD (48 residues), extends to about 8 nm and LL-37 (37 residues) forms a dimer with a nearly identical extension to DCD, since its N- and C-termini do not overlap. Both dimers are much larger than the typical membrane thickness (4–5 nm) so they can span the biological membranes. Due to the smaller number of residues, a Mag-2 dimer is considerably smaller but still long enough to span an average bacterial membrane.

Structural comparisons between wild-type Mag-2 and its variants provide valuable insights into the peptide’s conformational plasticity. While the NMR structure of wild-type Mag-2 in the presence of DPC (PDB 2MAG) is highly similar to our crystallographic structure (with a root-mean-square deviation (r.m.s.d.) of ~ 1 Å across atomic positions), it only displays a monomeric form (see Fig. S9A)^46^. Also, the structure of the S8A/G13A/G18A Mag-2 triple mutant, designed to reduce backbone flexibility, exhibited an almost identical dimeric arrangement to our crystal structure (r.m.s.d of 1Å for 44 Cα atoms). However, no higher oligomers were observed, likely due to the absence of detergents during crystallisation^50^ (PDB 4MGP, see Fig. S9C). Other NMR structures as the one for the F5Y/F16W double mutant (PDB 1DUM) in 0.5 mM DLPC (dilauroyl-L-a-phosphatidylcholine) revealed a Mag-2 dimer, but with significant structural deviations, in fact, the pronounced curvature of each monomer prevented accurate superposition onto our crystal dimer structure^78^ (see Fig S9B). Finally, two more Mag-2 dimer structures were obtained by using quasi racemic mixtures containing the D-form of Mag-2 derivative and L-peptide, in which one Ala was replaced by a β-amino acid residue (PDB 5CGO and 5CGN, see Fig. S9D)^51^. These structures differed from our dimeric arrangement primarily in the relative tilt of the monomers, with a root-mean-square deviation (r.m.s.d.) of 1.25 Å across 37 Cα atoms. In this work, we have shown how a dimeric form of wild-type Mag-2 can be stabilized by the presence of detergents (0.2% DPC) to render crystal structures (PDB 9HVN).

**Fig. 6:**
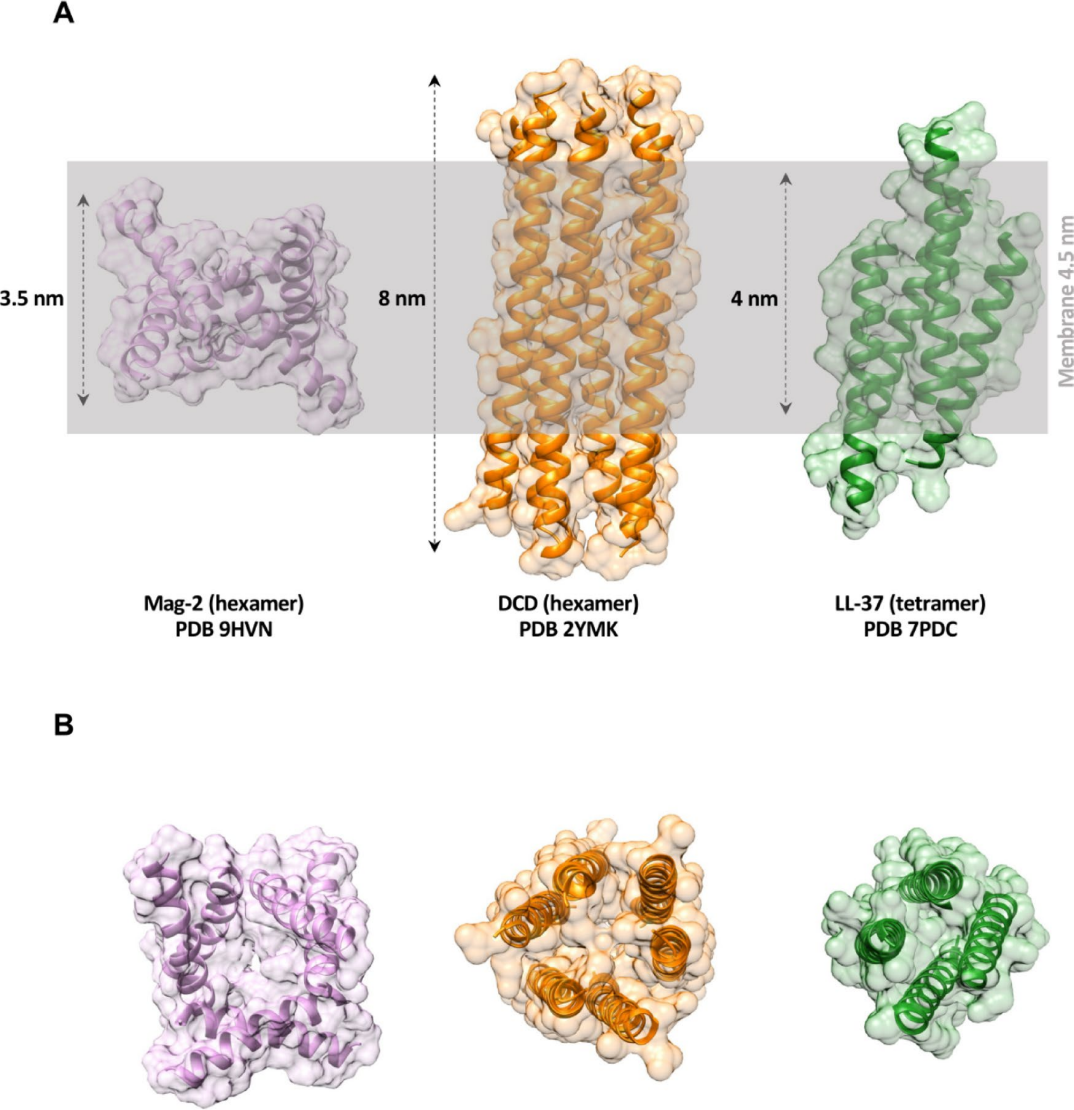
Structure of the three barrel-stave channels obtained by X-ray crystallography. (**A**) The hexamer of Mag-2 (purple), DCD hexamer (orange) and LL-37 tetramer (green) showing the monomers in ribbon and their transparent surface. Both, the DCD and LL-37 oligomers can span without any difficulties the length of the biological membranes (4–5 nm). The Mag-2 channel could still be embedded in the membrane, forming a funnel-like structure. (**B**) The top projection of the channels showing the width of the hole.

Beyond promoting folding, detergents and lipids also play a crucial role in increasing the likelihood of AMP oligomerisation. Analysing the crystal packing of Mag-2 revealed close distance connections of one dimer to additional Mag-2 dimers, suggesting the formation of the hexameric channel structure (see Fig. 2). The length of the two previously crystallised barrel-stave channels (hexameric for DCD and tetrameric for LL-37), allows them to span without any difficulty a biological membrane (see Fig. 6A). For the hexameric Mag-2 complex we revealed here, its height is only 3.5 nm, making it approximately 1 nm too small to span the membrane (see Fig. 6A). However, the complex could still be embedded in the membrane, forming a funnel-like structure, with lipid headgroups surrounding the channel borders. This could resemble melittin’s behaviour where oligomeric assemblies insert partially into membranes, disrupting lipid packing and creating localized membrane instability without requiring complete transmembrane spanning^39^. This model could explain why Mag-2 demonstrates antimicrobial activity despite structural limitations for full membrane traversal, suggesting that partial membrane insertion combined with local membrane disruption may be sufficient for bactericidal effects.

The barrel-stave channel formed by the Mag-2 hexamer embedded in a membrane bilayer, reveals a pore size of 0.7 nm. This is comparable to the inner diameter of human DCD in its hexameric form according to crystallographic data^18^ (see Fig. 6B). In addition, channel current conductance measurements done with magainin-1 (a closely related peptide differing by only two amino acids from Mag-2) revealed the presence of rigid pores in negatively charged membrane (PE/PG)^63^. The calculated mean of assembling monomers was 5.9 nm, in concordance with the crystallographic structure shown in this work. By different techniques, including Sum Frequency Generation (SFG) vibrational spectroscopy and Attenuated Total Reflectance – Fourier Transform Infrared spectroscopy (ATR-FTIR), Mag-2 has been shown to form dynamic toroidal pores with larger oligomerisation states^27,28,79^. Estimates of the Mag-2 toroidal pore´s stoichiometry range from four to ten monomers, with molecular dynamic simulations suggesting even 17 or 18 monomers^22,28,38,39,63^. The key question is whether Mag-2 can form both toroidal and barrel-stave channels, and if so, what factors determine which structure prevails. As a fast answer, membrane composition and peptide concentration appear to be critical in directing this process. As an example, the mode of action of F5W Mag-2 has been described as dual. By using confocal microscopy, two different Mag-2 pore sizes (diameter of ~ 2.8 nm and > 23 nm) have been described^80^. Recent simulations done by Tuerkova et al. suggest that Pro or Gly kinks in AMPs influence pore formation, promoting toroidal pores while disrupting barrel-stave structures^40^. This fact could explain the existence of an hexameric form of Mag-2 as observed in the crystal structure presented here. The constraints imposed by the crystal environment could play a role in restraining the flexibility of the Mag-2 kink, favouring the barrel-stave arrangement. In fact, no kink was observed in the Mag-2 monomers solved by crystallography in this work.

Although the dimer interfaces in the Mag-2 hexameric arrangement were relatively weak, the structure appeared plausible. To further assess its stability, we tested the hexamer in lipid membranes (POPE: POPG) using molecular dynamic simulations. The results confirmed that the hexameric structure remained stable under the chosen conditions and exhibited a remarked electrostatic field, facilitating spontaneous ion flow across the membrane pore. Specifically, we demonstrated the ability of the Mag-2 channel to allow the pass of chloride anions, being non-permeable to potassium cations. This behaviour aligns with the cationic nature of the inner part of the channel which is rich in lysine residues. The only negatively charged amino acid on the structure is located on the exterior part of the channel. These findings support the idea that Mag-2 can form an anion-selective channel, consistent with previous studies that observed a similar selectivity for Magainin-1 in POPC: DOPE (7:3) vesicles^81^. However, the predicted anion selectivity contrasts with electrophysiological data shown by Cruciani et al., who demonstrated that Mag-2 forms weakly cation-selective channels in planar lipid bilayers with a 5:1 selectivity ratio for monovalent cations over anions^82^. The authors explained this cationic selectivity by the inclusion of negatively charged lipids in the channel complex, contrasting with our barrel-stave structure that contains no lipids within the channel itself, resulting in a predominantly positively charged pore. Additionally, the POPE/POPG membrane composition used in our MD simulations may favour different selectivity properties compared to the experimental lipid systems used by Cruciani et al. These discrepancies highlight important questions about how membrane environment influences channel selectivity and will be addressed in future research by using electrophysiology or liposome leakage assays.

The observed chloride selectivity of Mag-2 channels suggests a mechanism of bacterial killing in which ion flow disrupts cellular homeostasis and contributes to cell death, as suggested for other AMPs. For example, the calculated chloride flow rate of 54 ions per 100 ns per channel could disrupt cellular ion homeostasis when multiplied across multiple channels. This value is consistent with the one observed in a protegrin octamer molecular dynamics simulation, in which 55 chloride ions traversed the pore during the 100 ns of simulation^83^. Additionally, bacterial cells maintain strict K^+^/Na^+^ gradients and pH balance, and disruption by selective anion flow could easily collapse membrane potential and ATP synthesis. Future studies will be necessary to definitively establish the relative contributions of selective ion transport versus general membrane disruption to the antimicrobial mechanism.

AMPs display different modes of action but due to their cationic nature they will contact the outer membrane as a primary target (in particular LPS), and will form pores in the inner membrane. Finally, the positive peptides will interact in the cytoplasm with negatively charged molecules such as ribosomes, RNA molecules or negatively charged proteins^13,84^. In this work, we used cryo-electron microscopy to visualize peptide-cell interactions. The very first electrostatic interaction of Mag-2 with the cell is mediated by peptide-outer membrane interactions. It has been described for LL-37, SMAP-29 and other peptides that due to their positive charges, LPS (and LTA in Gram-positive bacteria) are their main targets. Several LPS-AMP structures have been described by NMR such as cecropin P1 or pardaxin^85,86^.

Due to the strong positive charge of Mag-2, an interaction with LPS and phospholipids is highly probable. Indeed, EM images show significant alterations to both the inner and outer membranes upon Mag-2 interaction. Atomic Force microscopy further supports this, revealing that at low Mag-2 concentration, the peptide induces surface-bound vesiculation^87^. Among the changes that can be observed in the electron micrographs, the holes in the outer membrane did not exhibit a defined size. This correlates with the plethora of oligomers that magainin-2 can form depending on the concentration and membrane composition. Irregular holes formed in the OM have also been reported when using minicells in the presence of melittin or the *de-novo* designed AMP pepD2M^88^. The phenotype observed in the membranes of *E. coli* after incubation with Mag-2 differs from that previously reported for SMAP-29 or Dermcidin, where the most significant alterations were in the inner membrane, while the outer membrane remained largely intact^23^. In contrast, the phenotype observed with sub- or near-MIC LL-37 concentrations mirrors the phenotype seen with sub-MIC Mag-2 concentrations, characterised by damage to both membranes and the formation of stress vesicles^23^.

The Mag-2 channel structure is the third barrel-stave structure we have published. While both Mag-2 and DCD showed an hexameric arrangement, LL-37 channel was composed of four peptides^18,23^. Despite we cannot entirely rule out the possibility that the Mag-2 hexamer is a crystallisation artefact, its structure fits the mean assembling number observed by Watanabe and Kawano for magainin-1^63^. This inevitably raises several intriguing questions. Could the Mag-2 hexamer represent an intermediate state in membranes, restricting kink flexibility and promoting beta-barrel channel formation? Might this hexamer serve as a nucleation point for assembling higher-order structures, such as large toroidal pores? Could it facilitate the self-assembly of other magainin peptides in a manner similar to amyloid peptides^89^? Could exist cooperative pore formation mechanisms, including the formation of Mag-2/PGLa mixed hexamers? While further experiments are needed to explore these possibilities, the crystallographic Mag-2 hexamer demonstrates the potential of this peptide to form medium size channels capable of ion transport.

The coexistence of multiple pore mechanisms (barrel-stave vs. toroidal) depending on concentration and membrane composition adds complexity to structure-activity interpretations. Future electrophysiological studies using patch-clamp or planar lipid bilayer techniques will be essential to definitively characterize the ion conductance, selectivity, and kinetic properties of wild-type and mutant channels, thereby bridging the gap between structural data and dynamic functional behaviour in physiologically relevant membrane systems. These future investigations will provide valuable insights for translating our structural insights into a comprehensive understanding of Mag-2’s mechanism of action.

## Materials and methods

An overview of the experimental workflow is provided in Supplementary Figure S10.

### Peptides, lipids and detergents

Magainin-2 was chemically synthesized and purchased from BioServUK (https://bioservuk.com/) at purities higher than 95%. DPC was purchased from Affymetrix.

### Characterization of peptides by circular dichroism (CD)

40 µM magainin-2 in 10 mM phosphate, pH 7, was used for the secondary structure determination using a CD JASCO J-1500 spectrophotometer (Jasco Spectroscopic Co. Ltd., Hachioji City, Japan). Magainin-2 was analyzed at 20 °C and a scanning speed of 100 nm/min with a band width of 1 nm. All spectra were recorded at 0.5 nm resolution and data were reported as differences in molar absorption. The samples were measured in quartz precision cuvettes with a path length of 1 mm. DPC was added at 0.14 mM − 5.7 mM concentrations.

### Crystallisation and crystallographic studies of magainin-2

Crystallization of the hexameric channel was conducted with peptide-detergent mixtures (10 mg/ml mag-2, 0.5% DPC) in a 2 mM sodium phosphate pH 6.8 buffer, using the commercial screens from Jena Bioscience, Qiagen and Molecular Dimensions. Sitting drops of 400 + 400 nl (peptide + reservoir) were prepared by a Mosquito robot (TTP Labtech) and the progress of crystal formation was monitored using the Formulatrix Rock imaging system. Crystals were mounted from crystallization drops and data were collected at the synchrotron source SLS (Swiss Light Source, Villigen, Switzerland—beamline PX10). Data were recorded on the Pilatus detector 6 M (Dectris) at 100 K with beam attenuation of 20–50% at beamline PX10. Data were processed using the XDS/XSCALE program^90^. The structure was determined by molecular replacement using the monomeric NMR structure (PDB-entry: 2MAG) as a search model. The structure was refined by Refmac^91^, Phenix^92^ and BUSTER^93^ and manually modelled using the COOT program package^94^. The geometry of the structure was validated using the Molprobity server (https://molprobity.biochem.duke.edu/*).* All refinement and model statistics are given in Table S2.

### Molecular dynamics

Explicit solvent all-atom Molecular Dynamics (MD) simulations were performed with GROMACS v2016.5^95^. The magainin-2 hexameric structure was embedded in a phosphatidyl ethanolamine/phosphatidyl glycerol (POPE/POPG) membrane bilayer (3:1 ratio composition). The initial coordinates of the Mag-2 hexamer were directly taken from our crystal structure. The initial coordinates of the membrane bilayer were generated with MEMBRANE BUILDER using the CHARMM-GUI webserver^96,97^. The Mag-2 hexamer was placed into the membrane with an initial orientation in which the tiny pore is perpendicular to the membrane plane. Clashing lipids were initially removed from the system. Finally, a structural model of the Mag-2 hexamer embedded in a bi-layer lipid membrane composed of 144 POPE molecules and 48 POPG was obtained. The system was immersed in a cubic box of 8.08 × 8.08 × 10.4 nm^3^ filled with explicit water molecules (12527 molecules). Counter-ions (K^+^ and Cl^–^) were added to neutralize the total charge of the system at a final concentration of 1 M to mimic experimental conditions (514 ions in total). Equilibration of the whole system was achieved following the protocol described in^96^. The simulations were performed using the CHARM36 forcefield for peptide and lipids parameters^98^ combined with CHARMM TIP3P water model. Periodic boundary conditions were applied in the three spatial directions. All simulations were performed with all bonds involving hydrogen atoms constrained by means of the LINCS algorithm^99^, and a simulation time step of 2 fs. The Verlet cut-off scheme^100^ was applied to treat long-range non-bonding interactions. Explicit van der Waals and Coulomb potentials were used up to a cut-off distance of 1.2 nm. All simulations were performed under the NPT ensemble at 303 K and 1 bar by coupling to the Nosé-Hoover thermostat^101^ and Parrinello-Rahman barostat^102^. Five independent replicates of the system were simulated for 100 ns each replica. One of the replicas was extended for 200 ns to further check system stability. The system coordinates evolution of all molecular dynamic simulations was visualized and analyzed with VMD^103^ and GROMACS tools^95^. Average membrane conductance was estimated by dividing the ionic current by the potential difference across the membrane. The ionic current was evaluated as the number of ions passing through the channel per unit time. The potential across the membrane axis is calculated by first summing the charges per slice (every 0.12 nm) and then integrating twice of this charge distribution.

### Preparation of bacterial cells treated with AMPs for cryo-EM and data collection

*Escherichia coli* K12 cells were grown at 37 °C at 200 rpm to an OD600 of 0.6. Magainin was added to 1 ml cultures to reach a final concentration of 25 µM and cultures were further incubated at 37 °C while shaking at 300 rpm (Eppendorf Thermomixer, Hamburg, Germany). After 1 h, 100 µl of the samples or controls without AMPs were taken and cells were pelleted at 3000*g for 10 min at 4 °C. The cell pellets were resuspended using 20 µl of culture solution in order to concentrate the sample and reach a sufficiently high cell density for the visualization in the microscope.

To prepare vitrified grids for cryo-EM experiments, 4 µl of the concentrated sample solution in the case of the experiments with *E. coli* cells or from the vesicles incubated with LL-37 or LL-37 labeled with nanogold were applied onto glow-discharged Quantifoil R 2/1 200-mesh holey-carbon grids. Later the grids were blotted with a paper filter and were abruptly plunged in a liquid ethane bath, cooled with liquid nitrogen to − 196 °C, using a Vitrobot (FEI). Vitrified grids were cryo-transferred into a 626 DH cryo transfer holder (Gatan Inc.) and manually analyzed on a JEM-2200FS/CR (JEOL, Ltd.) transmission electron microscope equipped with a field emission gun (FEG) operated at 200 kV. No-tilted zero-loss two-dimensional (2D) images were collected under low-dose conditions, with a total dose of the order of 10–20 electrons/Å2 per exposure, on a 4 K × 4 K 15 μm pixel Ultrascan4000™ CCD camera (Gatan Inc.), at defocus values ranging from 2.0 to 4 μm. The in-column Omega energy filter of the microscope helped to record images with improved signal-to-noise (SNR) ratio by zero-loss filtering, using an energy slit width of 15 eV centered at the zero-loss peak of the energy spectra. Digital images were recorded using DigitalMicrograph™ (Gatan Inc.) software at different nominal magnifications, between 30,000 × and 60,000 ×, resulting in a final pixel size between 3.6 Å/pixel and 1.7 Å/pixel respectively.

## Supplementary Information

Below is the link to the electronic supplementary material.

Supplementary Material 1

## Data Availability

The atomic coordinates and structure factors have been deposited in the Protein Data Bank under accession code [PDB 9HVN]. Relevant data concerning the molecular dynamics simulations are available for download from Zenodo, DOI: 10.5281/zenodo.1596295.
