# Supplementary material for: Structure of a barrel-stave pore formed by magainin-2 reveals anion selectivity and zipper-mediated assembly
Source: Sci Rep. 2025 Nov 13;15:39830. doi: 10.1038/s41598-025-23539-1 (PMC12615594; doi:10.1038/s41598-025-23539-1)
Supplement: Supplementary file 1 — Supplementary Material 1 [file 41598_2025_23539_MOESM1_ESM.pdf]

Supplementary Information

Structure of a barrel-stave pore formed by magainin-2 reveals anion selectivity and zipper-mediated assembly

Enea Sancho-Vaello[a,*], Harun Kücükyildiz[b], David Gil-Carton[c,d,e], Xevi Biarnés[f], Kornelius Zeth[b,g,*]

[a] Department of Biochemistry and Molecular Biology, Universitat Autònoma de Barcelona, 08193 Cerdanyola del Vallès, Spain

[b] Department of Science and Environment, Roskilde University, Universitetsvej 1, 4000 Roskilde, Denmark.

[c] Basque Resource for Electron Microscopy, Biofisika, Leioa, Spain.

[d] Ikerbasque, Basque Foundation for Science, Bilbao, Spain.

[e] CIC bioGUNE, Derio, Spain

[f] Laboratory of Biochemistry, Institut Químic de Sarrià (IQS), University Ramon Llull (URL), 08017, Barcelona, Spain.

[g] Faculty of Biology, University of Regensburg, Universitätsstraße 31, 93053 Regensburg, Germany.

* Co-corresponding authors:

Kornelius Zeth
Biology, University of Regensburg
Universitätsstraße 31
93053 Regensburg, Germany
Kornelius.Zeth@biologie.uni-regensburg.de

Enea Sancho-Vaello
Department of Biochemistry and Molecular Biology
Biosciences Faculty, Building C, Office C2/427,
Campus UAB, 08193 Bellaterra, Spain
enea.sancho@uab.cat

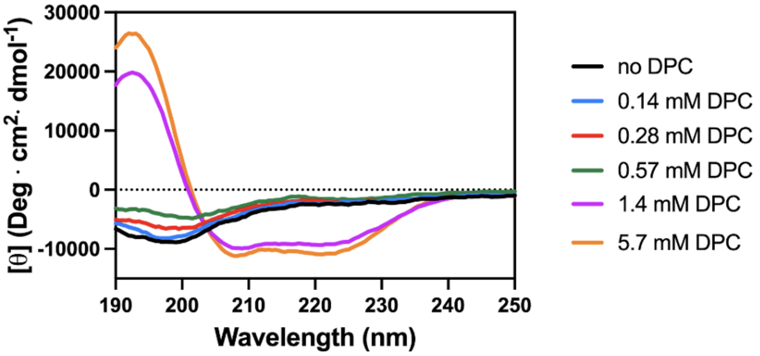

Figure S1 – ***CD spectra and cross-linking of Mag-2 in the presence of increasing amounts of DPC.*** Increasing concentrations of DPC (0.14 mM blue line, 0.28 mM red line, 0.57 mM green line, 1.4 mM purple line, 5.7 mM orange line) were added to a solution of 40 µM Mag-2 (black lines) and the folding process was monitored. Mag-2 exhibits a disordered structure in the presence of 10 mM phosphate buffer, pH 7, at concentrations of DPC below the CMC. Only in the presence of high concentrations of DPC, the spectrum shows the characteristic bands of α-helical conformation. The curves are based on triplicate measurements and the buffer background was subtracted.

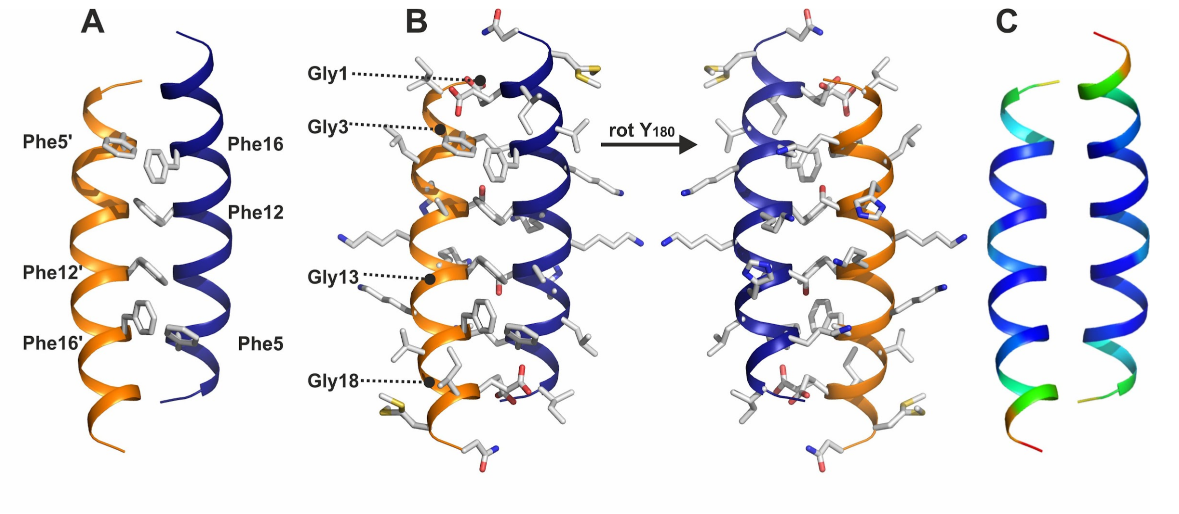

Figure S2 – Dimeric structure of Mag-2 emphasizing important structural elements. (A) Each Mag-2 peptide monomer in cartoon representation (colour coded orange and blue) contains three well-structured Phe residues (marked Phe5/Phe5', Phe12/Phe12', Phe16/Phe16') highlighted in stick representation. In the dimer these residues form a Phe-Zipper arrangement essentially responsible for the monomer-monomer interactions. **(B)** Dimer structure of Mag-2 with all side chains shown in stick representation and the residue numbers assigned according to the sequence. There are four glycine residues (Gly1, Gly3, Gly13 and Gly18) leading to an increased flexibility of the structure in the N- and C-terminus. **(C)** Backbone representation of Mag-2 using the B-factors of Ca atoms to assign structural flexibility. Red and orange indicate higher B-factors (high flexibility) while blue and green colours indicate lower B-factors. The structural flexibility becomes more evident at the termini.

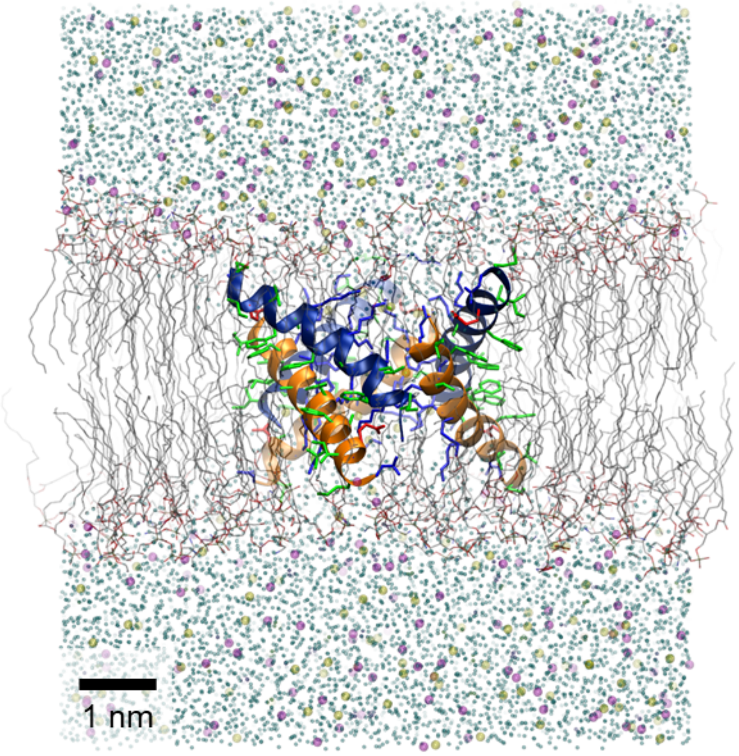

Figure S3 – Initial coordinates of magainin-2 channel embedded in the POPE/POPG membrane bilayer. The hexameric structure is shown in ribbons and solvent accessible surface colour coded according to the amino acid residual properties: hydrophobic residues are coloured in green, positively charged residues are blue and negatively charged residues are red and amphiphilic residues are marked in magenta. Membrane lipids are represented as thick lines and coloured by atom type (C: black, O: red, N: blue). Solvent molecules are represented as small spheres (water in cyan, chlorine ions in yellow and potassium ions in pink).

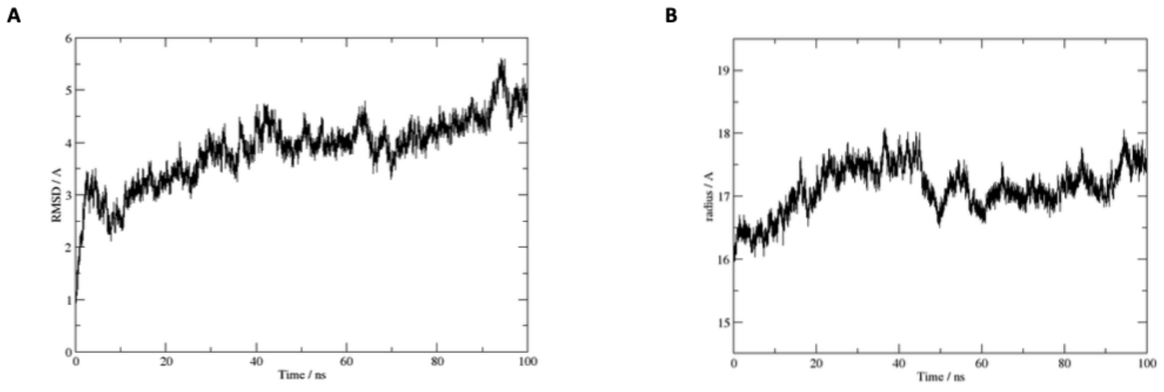

Figure S4 – Evolution of the **(A)** root-mean squared displacement (RMSD) and **(B)** radius of gyration of the hexameric magainin-2 channel along the membrane-embedded simulation. The RMSD evaluates the overall structural stability of the hexamer with respect to the initial coordinates. The trend in RMSD indicates a rather stable system, with an average displacement of 0.4nm with respect to the initial structure of the hexamer as a consequence of the thermal equilibration in explicit membrane and solvent. This structural displacement is translated into an increase of the radius of gyration (from 1.6nm to 1.7nm).

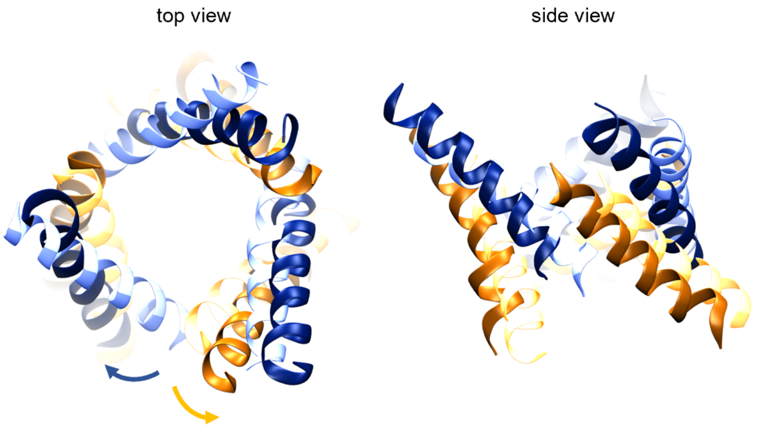

Figure S5 – Structural superposition of the hexameric magainin-2 channel structure at the start (light colours) and the end (dark colours) of the MD simulation. Peptides coloured as in Figure 2B and 3A. Arrows indicate the direction of the helix tilt.

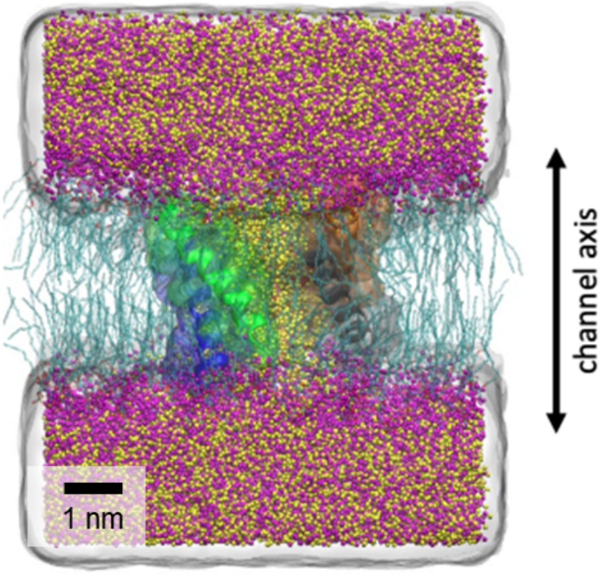

Figure S6 – Density of solvent molecules (water and ions) across the system. All positions were sampled by chloride ions (yellow spheres) and potassium ions (pink spheres) during the whole simulation. Side-view. Membrane lipids are represented as lines and coloured by atom type. The hexameric magainin-2 channel is in the middle. Each peptide is coloured differently and represented as ribbons and solvation surfaces. The solvent is represented as a white cubic volume. Explicit water molecules, present during the simulation, are omitted for clarity.

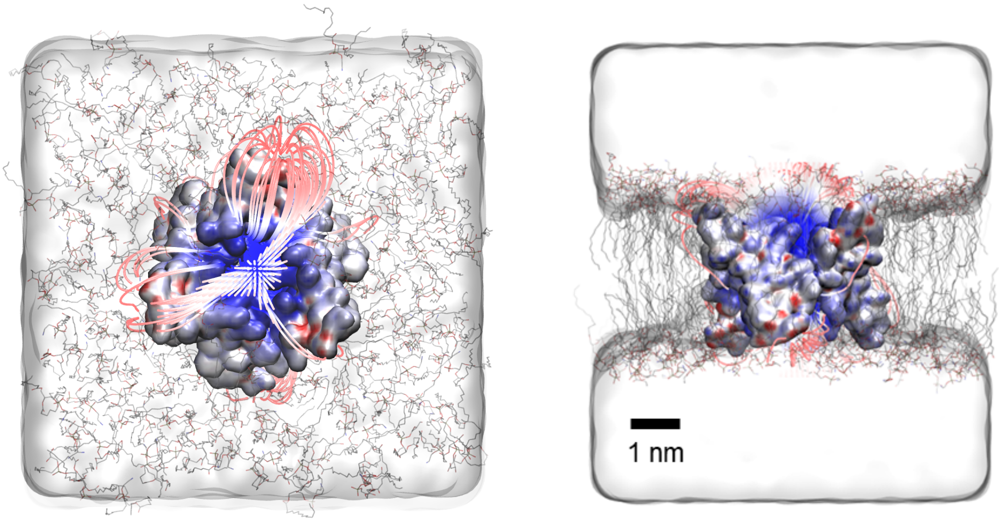

Figure S7 – Electrostatic potential of Mag-2 hexamer projected onto the protein surface. Left: top-view, right: side-view. Colour scale from -30 eV (red) to +30 eV (blue). Membrane lipids are represented as thick lines and coloured by atom type (C: black, O: red, N: blue). Solvent is represented as a grey cubic volume. Continuous electrostatic potential field is represented as thick lines coloured according to the electrostatic potential value (from 0 eV, red to 20 eV, blue).

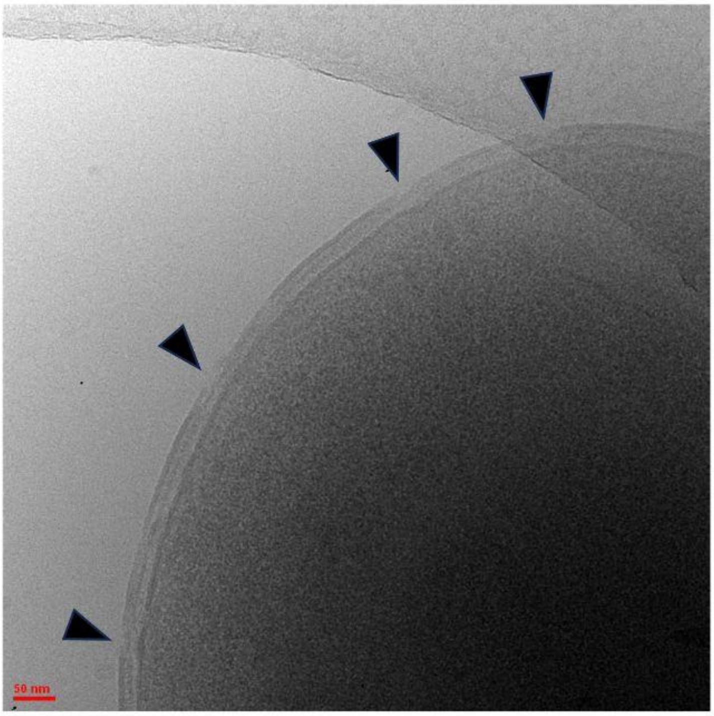

Figure S8 – Cell wall disruption of E. coli cells induced by Mag-2 imaged by cryo electron microscopy. *E. coli* K12 cells were incubated with Mag-2 at concentrations of 25 µM slightly above the MIC and imaged under the same conditions as the untreated cells. The outer membrane shows clear hole-like structures (see black triangles).

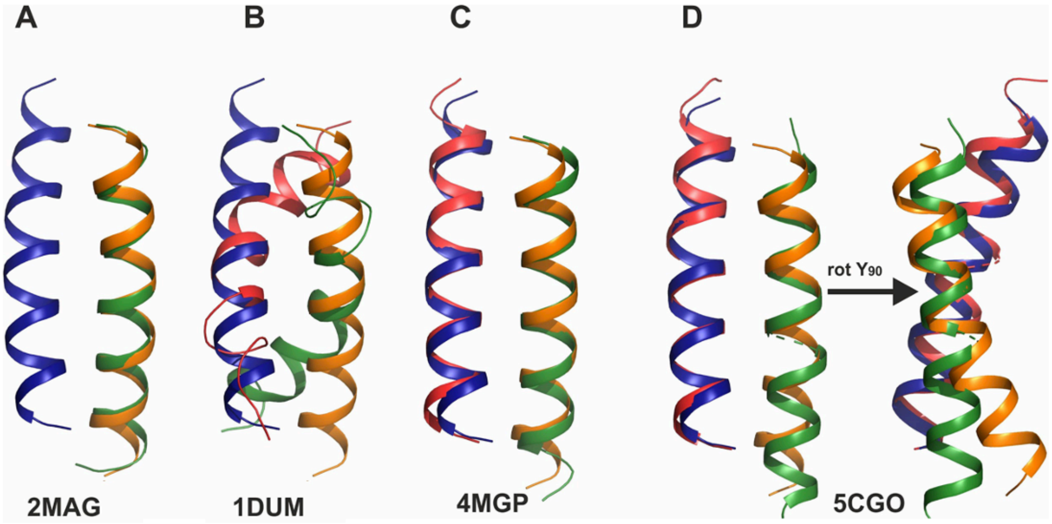

Figure S9 – Superposition of previously determined magainin structures with atomic resolution structure of Mag-2. (A) The Mag-2 structure in orange/blue is superimposed onto the monomeric magainin-2 structure solved by NMR marked in green. The rmsd of the two structures is 1 Å (for 44 Cα atoms) (PDB 2MAG) [46]. **(B)** The Mag-2 crystal structure in orange/blue is superimposed onto the dimeric Mag-2 mutant structure solved by NMR. The rmsd of the two structures is 2.7 Å (for 44 Cα atoms) (PDB 1DUM) [80]. **(C)** The Mag-2 structure in orange/blue is superimposed onto the monomeric Mag-2 mutant structure (PDB 4MGP) [50]. The rmsd of the two structures is 0.6 Å (for 44 Cα atoms). **(D)** The Mag-2 structure in orange/blue is superimposed onto the monomeric Mag-2 structure solved by NMR (PDB 5CGO) [51]. The rmsd of these two structures is 1.25 Å (for 37 Cα atoms). In D, the structure superposition is shown from two angles related to each other by a 90 degrees rotation around the Y-axis.

Mag-2 Peptide Synthesis & Purification
↓
Circular Dichroism Analysis
(DPC concentration optimization: 0-5.7 mM)
↓
Crystallization Screening
(Commercial screens + 0.2% DPC)
↓
Crystal Optimization & Data Collection
(Synchrotron radiation, 1.05 Å resolution)
↓
Structure Determination
(Molecular replacement using PDB 2MAG)
↓
Molecular Dynamics Simulations
(POPE/POPG membrane, 100 ns, GROMACS)
↓
Bacterial Cell Treatment & Cryo-EM Analysis
(E. coli K12 + 25 μM Mag-2, 1 hour)

Figure S10 – Workflow overview of the experimental procedures employed in this study.

Tables

Table S1 - Aromatic - Aromatic interactions within 4.5 and 7 Angstroms

Position	Residue	Chain	Residue	Position	Chain	D (centroid – centroid)	Dihedral Angle
5	PHE	A	12	PHE	H	5.82	152.38
5	PHE	A	16	PHE	H	5.35	129.22
12	PHE	A	12	PHE	H	4.82	171.41
12	PHE	A	5	PHE	H	5.82	152.74
16	PHE	A	5	PHE	H	5.35	129.31

Table S2 - Data collection and refinement statistics (molecular replacement)

**Space group**	P 63 2 2
**Cell dimensions**	
**a, b, c (Å)**	34.359, 34.359, 55.795
**α, β, γ (°)**	90, 90, 120
**Resolution (Å)**	29.76-1.05 (1.077-1.050)
**R_{sym} or R_{merge}**	0.029/0.92
**CC* in outermost shell**	51.9
I*/ σ*I	27.36 / 1.93
**Completeness (%)**	97.81
**Redundacy**	8.1/4.35
**Refinement**	
**Program REFMAC**	
**Resolution (Å)**	29.76-1.05
**No. reflections**	8509
**R_{work}/R_{free}**	0.17 / 0.18
**No. atoms**	
**Protein**	192
**Water**	10
**Carbonate**	0
**B-factors**	
**Protein**	22.4
**Water**	45.3
**R.m.s. deviations**	
**Bond lengths (Å)**	0.014
**Bond angles (°)**	1.553
**Ramachandran statistics**	
**Residues in favoured region No (%)**	100
**Residues in allowed region No (%)**	0
**Residues in outlier region No (%)**	0
**PDB-entry**	9HVN
**Crystallization conditions**	

*Values in parentheses are for highest-resolution shell.
